# New enrollment under the affordable care act: leading the way for community health centers in Southern California

**DOI:** 10.1186/s12913-018-3469-z

**Published:** 2018-09-21

**Authors:** Omar Viramontes, Michael Hochman, Martin Lee Serota, Elvia Delgado, Gerardo Moreno

**Affiliations:** 1 UCLA Health and Department of Medicine, David Geffen School of Medicine at UCLA, 10880 Wilshire Blvd, Suite 1800, Los Angeles, CA 90024 USA; 2 Gehr Family Center for Implementation Science, Department of Medicine Keck School of Medicine, University of Southern California, 2020 Zonal Ave. IRD 320, Los Angeles, CA 90033 USA; 3 AltaMed, Inc., 2040 Camfield Ave, Los Angeles, CA 90040 USA; 4 Department of Family Medicine, David Geffen School of Medicine at UCLA, 10880 Wilshire Blvd, Suite 1800, Los Angeles, CA 90024 USA

**Keywords:** Community health centers (CHC), Federally qualified health centers (FQHCs), Promotoras, Affordable care act (ACA) enrollment

## Abstract

**Background:**

The Affordable Care Act (ACA) has improved healthcare access in the community health centers that have played a critical role in enrolling low income and minority patients. This study examined the ACA enrollment for one of the largest federally qualified community health centers in the country.

**Methods:**

An exploratory sequential mixed method study was used as the main qualitative and quantitative approach for this study. Key stakeholders (*n* = 6) were interviewed as part of the qualitative component, and information about barriers and best practices were acquired. As part of the quantitative analysis, we examined cross-sectional data among 59,272 AltaMed enrollees in 2013–2015. We analyzed data on age, gender, language, ethnicity, and enrollment periods. The interviews were conducted first and followed by the data analysis.

**Results:**

AltaMed was the top enroller of patients in ACA insurance plans in California (2013–14 and 2014–15) through the state exchange and Medicaid expansion. Using key stakeholder interviews, 5 main barriers were identified and 5 innovative solutions that allowed AltaMed to enroll people into the state exchange and Medicaid expansion. Barriers to enrollment included training, new workflows, and enrollment of Young Invincibles, and these enrollment barriers were overcome with community health workers.

**Conclusion:**

Enrollment barriers were overcome through AltaMed’s community-based approach and long term community partnerships.

## Background

Community health centers (CHCs) provide access to health care for underserved communities and vulnerable populations [1]. Health centers cared for approximately 21.7 million people in 2013, 62% of whom were members of ethnic and minority groups, and 35% of whom had no health insurance. The Affordable Care Act (ACA) established the Community Health Center Fund to provide $11 billion over a five-year period to support the operation, expansion and construction of health centers [2]. Due to the increased funding, CHCs grew significantly over the last few years since the implementation of the ACA. In 2017, CHCs provided care to 1 in 13 people in the US, 1 in 6 people receiving Medicaid, 1 in 3 low income uninsured and 1 in 3 individuals living below the federal poverty level [3, 4].

The ACA expanded access to care through both Medicaid expansion as well as the creation of state marketplaces to facilitate the purchase of commercial insurance with subsidies for low income individuals. In 2015, 19 states and the District of Columbia chose to manage these marketplaces themselves (California was one such state) [3, 5].

Furthermore, the ACA reinforced the importance of having community health workers (CHWs), or *promotoras,* conducting outreach in the communities served. Recognizing the value of CHWs, the ACA authorized the Centers for Disease Control and Prevention (CDC) to issue grants to organizations, yet a major hurdle was that Congress did not appropriate funds for these grants. Nevertheless, the ACA has reinforced the value of CHCs in the US health system [6]. Many organizations, including an Agency for Healthcare Research and Quality (AHRQ) review found that CHWs are essential to securing access to health care, coordinating timely access to primary care and preventive services, and helping individuals manage chronic conditions [7]. Many CHCs – which have used community health workers in many capacities in the past – employed these team members to promote enrollment in the new insurance options offered through the ACA [5, 8].

With the passage of the ACA many organizations at the local, state and national stage undertook the challenge of enrolling people in insurance plans by using CHWs.. A study of California hospitals suggested adopting the following 8 strategies to enrolling patients for healthcare coverage [9]. It suggested designing effective and transparent enrollment practices, determining optimal levels of staffing, positioning staff at key access points, educating patients on insurance options, using innovative strategies to reach vulnerable populations, partnering with external stakeholders and service vendors, and finally assessing the environment pre and post each enrollment period effort [9]. A different but similar approach was used by Nebraska’s AIDS Drug Assistance Programs (ADAP), which developed an ACA enrollment strategy with the input of stakeholders across the state [10]. Using program case managers they identified individuals that were greater than 18 years of age, met the ADAP eligibility guidelines and ACA eligibility guidelines [10]. Health Communication Research Laboratory analyzed data pooled across 15 years and suggested the following recommendations to effectively target low-income and racial/ethnic minority groups [11]. They included 12 action steps which covered making partnerships, working on outreach, developing clear messages and training messengers, identifying the priorities of vulnerable populations and ensuring health information was clear and trusted [11]. Overall, the literature shows that the most successful enrollment strategies include a combination of case managers, newly trained personnel or CHWs equipped with the knowledge and tools to enroll patients post-ACA.

AltaMed, the largest community health center in Southern California, with over 40 locations provides care to thousands of patients with the help of 200 primary care providers and 1800 community specialists. AltaMed opted to utilize community health workers – which they refer to as health promoters – for their ACA enrollment efforts. During the first 2 years of insurance expansion under the ACA, AltaMed enrolled more patients in Medicaid and the commercial exchange in California than any other certified entity in California, and more than any other CHC in the nation. In this field action report, we describe the enrollment trends, and the challenges and solutions learned that helped establish best practices at AltaMed, the largest community health center in the U.S.

### Setting

AltaMed has over 40 clinic locations and provides adult and pediatric primary care, dental, adult day health, behavioral health, and HIV services. AltaMed opted to provide free in person enrollment assistance to its community members because a large portion of the population they serve had the potential to benefit from insurance expansion under the ACA. In preparation for coverage expansion, AltaMed opened two Health Insurance Resource Centers (HIRCs) or enrollment sites, one in Commerce, CA, and the other in Santa Ana, CA, where bilingual (Spanish and English) enrollment counselors (i.e., *promotoras*) provide free and confidential health insurance enrollment assistance.

### Enrollment program description

Before the implementation of the Affordable Care Act, AltaMed was enrolling community members into Medicaid, Medicare, HWLA (Healthy Way LA), CHIP (Children’s Health Insurance Program), and other smaller state and county programs within their usual service sites (i.e., clinics). HWLA is a free public health care program available to underinsured or uninsured, low income residents of Los Angeles County. Most of the enrolling process was done through paper applications with the support of AltaMed’s patient care coordinators. AltaMed also engaged in community efforts, including establishing store front events and community enrollment events, and in the process developed a trusting relationship with the community.

After ACA implementation, the strategy changed. The ACA expanded eligibility criteria for subsidized insurance and made it a requirement that everyone obtain coverage. AltaMed targeted enrollment efforts to their 65,000 existing patients who were deemed eligible for coverage in 2013. AltaMed invested $1.7 million in discretionary funds during the first year of the ACA for a multi-channel advertising campaign, storefront outreach, and other outreach efforts to promote enrollment. During the ACA’s second year, AltaMed invested $455,000 on advertising (billboards, digital marketing and radio) to reinforce their presence to community members. Furthermore, AltaMed trained 150 cross-functional staff as certified enrollment counselors (CECs) during the first year of ACA enrollment efforts. The staff members were full-time AltaMed employees who also became part-time enrollment counselors. AltaMed’s approach to training new CECs was designed to meet the increased demand during the first ACA enrollment period (October 1–March 31). AltaMed also created a “Lean Team” to enhance the efficiency of the enrollment processes.

During the second year, AltaMed created a “Core Team” that was fully trained to handle all components of the enrollment application. Twenty-five *promotoras* were fully trained to manage patient education, program applications, renewals, and primary care provider (PCP) changes at the Health Information Resource Centers and other select clinics. The approach was to target uninsured and underinsured community members by having *promotoras* engage community members in different locations. For example, they organized community enrollment events, leveraged their media partnerships and had a presence at health fairs, 5 K races, and school sites, yet their primary efforts occurred at their two enrollment centers.

Enrollment efforts focused on enrolling patients in eligible health coverage. Patients could then choose their preferred health care provider within their coverage network – including either AltaMed or non-AltaMed providers.

## Methods

We conducted an exploratory sequential mixed method study [12, 13]. The qualitative component consisted of key stakeholder semi structured interviews and the quantitative component was a secondary data analysis of patients enrolled in insurance coverage during the first two enrollment periods of the ACA. The interviews were conducted first and followed by the data analysis.

### Qualitative stakeholder interviews

The medical director of innovation (MH) identified key senior leadership executives who were directly or indirectly responsible for overseeing enrollment. These stakeholders were then made available to the interview team. All stakeholders (*n* = 6) were part of the senior leadership in AltaMed, ranging from marketing executives, outreach directors, and medical directors. The interviewers gathered information around three main categories, 1) background information of stakeholder participants, 2) what where the main barriers their organization encountered, and 3) what were their respective departments and organizations best practices regarding patient enrollment. The interviews were conducted (OV) in person at AltaMed in the summer of 2015, and field notes were recorded for each interview.

The research team analyzed the interview and field notes using conventional content analysis methods [12, 14, 15]. Two interviewers (OV and GM) reviewed notes and discussed common themes. Five main themes were described in detail and framed into the five best practices. All stakeholders were then asked for additional input and agreed on the final version of the five main challenges and five best practices to enrolling patients in the ACA. The qualitative phase of this study was approved by the UCLA Human Research Protection Program (HRPR).

### Quantitative analysis

We examined data for 59,272 AltaMed enrollees between 2013 and 2015. Data on age, gender, language and ethnicity by enrollment periods was analyzed. This study was a secondary analysis with de-identified data, the University of California, Los Angeles Office of the Human Research Protection Program (OHRPP) reviewed the study and exempted this phase from further review.

Demographic variables included age, gender and language and ware derived from enrollment figures for both enrollment periods. Covered California plans were insurance plans from the state-exchange market in California, while Medi-Cal is California’s version of the federal program, Medicaid. Medi-Cal expansion was the expansion of Medicaid services as a result of the ACA. Each patient was stratified by insurance category. They included Covered California plans, Medi-Cal, Medi-Cal expansion, and Health Way LA (HWLA). While, Healthy Way LA is a Los Angeles County’s sponsored Medicaid expansion program. Furthermore, ethnicity/race and income were derived from the patient population at AltaMed and not from the initial enrollment forms. The ethnicity and race of patients was coded based on self-reported details by patients during clinic registration check in.

The enrollees were broken down into those anchored and non-anchored. Anchored patients were those who obtained their medical care at AltaMed while non-anchored patients were those who received care elsewhere. The ACA Enrollment Period 1 (EP1) went from October 1, 2013 to March 31, 2014 and Enrollment Period 2 (EP2) went from November 15, 2015 to April 30, 2015. Univariate frequencies, means, and histograms were calculated for all variables, and bivariate cross tabulations were performed.

## Results

Table 1 provides the demographic characteristics of enrollees at AltaMed using the community *promotora* model. There was a higher enrollment rate of females (60%) in both enrollment periods than males (40%). The majority of new enrollees fell under two age groups. One quarter of new enrollees were aged 19–29, while those 45–64 years old accounted for 35% of new enrollees. Combined, the two groups accounted for over 75% of new enrollees. Spanish-speaking populations were over represented among new enrollees, at approximately 56% during EP1 and 40% during EP2. Across the new enrollees, 37.5% were White Hispanics, 9% White Non-Hispanics, 2% African Americans, 2.5% Asian Americans and 49% unknown or other.

**Table 1:** Demographics of patients enrolled at AltaMed during the ACA enrollment periods

Patient Characteristics		Enrollment Period 1 (*n* = 19,231) %	Enrollment Period 2 (*n* = 15,591) %
Age	0–18	13%	19%
	19–29	21%	24%
	30–44	14%	18%
	45–64	45%	35%
	> = 65	7%	4%
Gender	Female	60%	59%
Language	English	31%	24%
	Spanish	56%	40%
	Unknown/other	13%	36%
Ethnicity/Race	White Non-Hispanic	9%	9%
	White Hispanic	37.5%	37.5%
	African American/Black	2%	2%
	Asian	2.5%	2.5%
	Other/Unknown	49%	49%
Income as Percentage of Poverty Level	100% and Below	57%	57%
	Over 101%	12%	12%
	Unknown	31%	31%

**Fig. 1:**
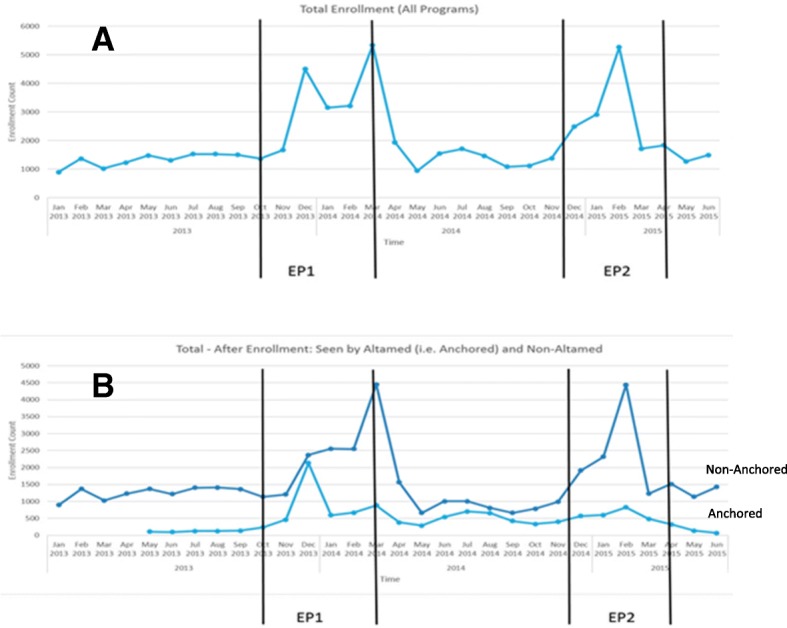
**a** Total enrollment for ACA enrollment periods and **b** anchoring and non-anchoring rates after enrollment

Figure 1 shows the number of enrollees for the past 3 years by month, and illustrates the increase in enrollment after the implementation of the ACA. Before the ACA, a steady enrollment rate of about 1500 per month was observed. After the ACA, we see a sharp increase in enrollment in both EP1 and EP2. During the EP1 enrollment period, 19,232 persons were enrolled with the highest enrollment rates in the months of December 2013 and March 2014. During EP2, 15,591 persons enrolled and the enrollment peak was in February 2015. Figure 1 also illustrates the number of anchored and non-anchored new enrollees at AltaMed in the last 3 years. During the late EP1 and throughout EP2, the anchoring rates did not keep up with the non-anchoring rates. In 2013, the numbers of anchored and non-anchored patients were 3420 and 15,992, respectively. In 2014, these increased to 6437 (anchored) and 18,935 (non-anchored). The most recent 2015 data shows 2433 anchored and 12,055 non-anchored patients.

Figure 1 illustrates significant peaks in both enrollment periods. During EP1, the first enrollment peak happened in December 2013. There was an additional peak in March 2014 because community members found that EP1 was actually scheduled to conclude at the end of March instead of February. During EP2, enrollment peaked in February 2015.

**Fig. 2:**
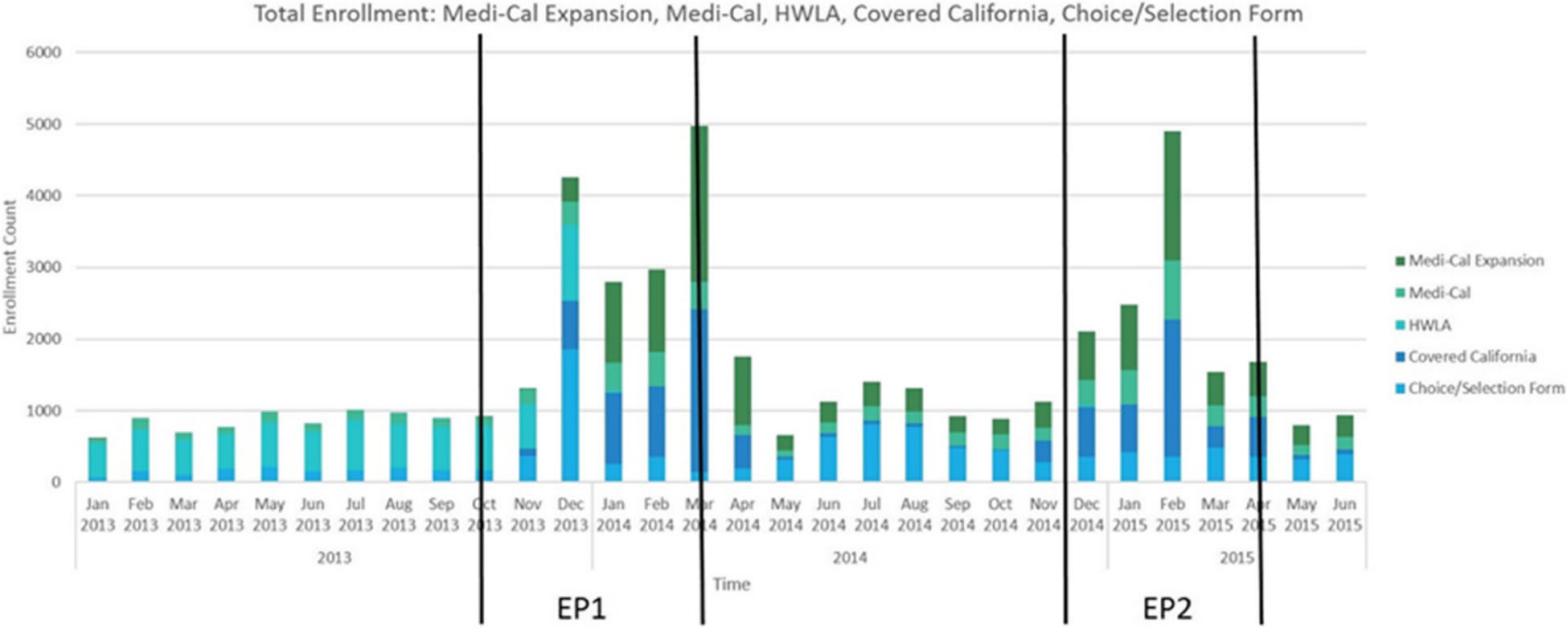
Enrollment in the top 5 insurance programs

Five plans represented 80% of those enrolled (Fig. 2). Medicaid expansion and Covered California plans are the largest insurance categories with the highest number of enrollees. Covered California is the online health insurance exchange in California, where enrollees can apply for health insurance and receive subsidies to offset the cost of coverage. Healthy Way LA (HWLA) enrollment decreased from EP 1 to EP2 and was eventually rolled into Los Angeles County’s Medicaid expansion program.

Table 2 lists the obstacles encountered during the implementation of ACA and describes the solutions to these challenges. The main challenges included not having a reporting infrastructure to track enrolled patients, the Covered California Training Module was insufficient to train people, balancing productivity versus delivering personalized care, a new healthcare insurance application was necessary to enroll patients, and a lack of desire to enroll by *Young Invincibles*, those uninsured between the ages of 18–34. AltaMed used collaboration among different departments to create an infrastructure to efficiently keep track of all enrolled patients. By acquiring key partners, AltaMed created and provided its own Supplemetal Training Model to train CHWs. These CHWs were then better able to serve previously uninsured populations, and ensure productivity and personalized care were at the top of their priorities when enrolling patients. AltaMed also partnered with AmeriCorps to enroll young college students at different California State Universities (CSUs) – mainly CSU Long Beach and CSU Los Angeles. Thus, AltaMed identified the key 5 barriers to enrollment and through collaboration, innovation and outreach they were able to produce 5 best practices at enrolling individuals.

**Table 2:** Top 5 challenges and solutions to increasing ACA enrollment per stakeholder interviews

Obstacles	Innovative Solutions
Lacked data/reporting infrastructure	Collaboration between different departments. The information technology (IT) department and enrollment teams built the necessary structure to gather the enrollment data of community members.
The Covered California Training Module was insufficient	AltaMed’s Supplemental Training Module – allowed enrollment counselors to be equipped to be deployed to the community. 1st Year: Lean Teams (each person was specialized in a few sections of the application). 2nd year: Core Team (each member handled the entire application)
Productivity vs. Personalized Care	A specific target time was given to enroll a person, but more time was provided if needed.
New Application/Procedure to enroll people into Healthcare insurance (i.e. Covered California)	Coordination was key: Staff (i.e. IT), trainings, enrollment events (e.g., Saturday events in middle schools, 5 k races) and other partners (e.g., churches, schools).
Young Invincibles (young college students)	AltaMed, jointly with AmeriCorps (i.e., young college graduates) utilized a combination of personal connections, the benefits of having health insurance, and outreach to enroll students and their families at CSU campuses.

## Discussion

The ACA has reinforced the value of integrating *promotoras* into interprofessional health care teams [16, 17] AltaMed jointly with community partners, utilized community engagement led by *promotoras* and successfully enrolled low-income community members, including many college students. The stakeholders overwhelmingly agreed that this was an effective and successful model. But the stakeholder interviews also identified many challenges that required community engaged solutions to ensure new enrollment during the ACA. To meet enrollment demands, all *promotoras* and free personnel worked up to 18 h days during the last 2 weeks of each enrollment periods. Stakeholder interviews identified p*romotoras* and other CHWs as essential enrollment team members due to their roles as patient educators and advocates for uninsured, minority and underserved populations [17, 18].

The demographic data showed an overrepresentation of White Hispanics (37.5%) compared to other racial/ethnic groups. This was due to the geographical location of clinics in East Los Angeles and Orange County which AltaMed held their enrollment events. Throughout Los Angeles County, White Hispanics make up 47% of the total population [19]. Moreover, there was a representation of women at 60% of the total enrolled patients.

AltaMed identified 5 main barriers for post-ACA enrollment, they lacked an infrastructure to gather and retain newly enrolled patients, the Covered California training module was insufficient, the new ACA enrollment application was new and convoluted, balancing productivity versus personalized care was a real challenge, and enrolling the young invincibles (young college students) was a tough challenge. Just like other community health centers and academic centers, AltaMed utilized partnerships with local organizations (e.g. church and community organizations) to enroll people directly in their communities, did targeted outreach to key groups such as young people, and identified and met the needs of vulnerable groups (e.g. low income groups) [10, 11]. Yet, unlike other initiatives and organizations, AltaMed was innovative by training key staff and equipping them with a personal AltaMed supplemental training module to make them proficient at enrolling patients. Just like other post-ACA initiatives, AltaMed identified some of the same barriers and created solutions in response to them. Yet, this paper illustrates some unique approaches and solutions. Thus, producing 5 best practices regarding patient enrollment under the ACA.

Despite having lower anchoring rates compared to non-anchoring rates, AltaMed made it their mission to enroll as many people as possible. AltaMed’s enrollment strategy was to enroll everyone regardless of whether or not they made AltaMed their medical home. Stakeholders noted the dense healthcare system in the Los Angeles area including multiple CHCs, private practices, and medical hospital centers as the main reason that contributed to the high non-anchored rates at AltaMed. The implications are significant for health care systems because some will opt to spend a great deal of resources in enrolling patients and connecting with them, while other systems will not. However, as the stakeholders pointed out, patients are comfortable with institutions that do early outreach and are able to help patients from the beginning of a process. Thus, trust and a quality reputation for connecting with the needs of the community are very important assets for any current and future successful health care system. Regardless, AltaMed as a public institution, realized early on that enrolling everyone is the best practice to help mitigate disparities in access to care. AltaMed’s enrollment best practices may benefit other community health centers by increasing their enrollment rates and better serving patients in underserved communities.

Key informant interviews revealed that the enrollment approach described has limitations. While enrollment rates were high at AltaMed, further work is needed to learn about the barriers and approaches to anchoring new enrollees to their own community health centers. The major challenge for many CHCs in future enrollment periods will be retention, and sustaining new enrollees. Stakeholders emphasized that finding the right number of resources to reach enrollment goals will be important.

Another systematic policy change for CHCs to consider is the recent repeal of the individual mandate that was included in the “Tax Cuts and Jobs Act” in December 2017 [20]. The ramifications are great as this repeal threatens to increase the risk pool by eliminating health insurance sign-up penalties for young and healthy uninsured individuals. CHCs should consider using the above mentioned community-based strategies to target new and subsequent enrollment in healthy and young individuals.

## Conclusions

Overall, during the first 2 enrollment periods of the Affordable Care Act, AltaMed enrolled a significant number of low income and minority patients. They were able to achieve this identifying major barriers and then creating innovative and targeted solutions. Overall, enrollment barriers were overcome using a community-based approach with a commitment to community partnerships.

## Abbreviations

ACA: Affordable care act
ADAP: AIDS Drug Assistance Programs
AHRQ: Agency for Healthcare Research and Quality
CECs: Certified enrollment counselors
CHCs: Community health centers
CHIP: Children’s Health Insurance Program
CHWs: Community health workers
CSUs: California State Universities
EP1: Enrollment period 1
EP2: Enrollment period 2
HWLA: Healthy way LA
OHRPP: Office of the Human Research Protection Program
PCP: Primary care provider
